# Reliability and Validity of the Persian Version of Compulsive Eating Scale (CES) in Overweight or Obese Women and Its Relationship with Some Body Composition and Dietary Intake Variables

**Published:** 2016-10

**Authors:** Seyed-Ali Mostafavi, Seyed Ali Keshavarz, Mohammad Reza Mohammadi, Saeed Hosseini, Mohammad Reza Eshraghian, Payam Hosseinzadeh, Maryam Chamari, Zeinab Sari, Shahin Akhondzadeh

**Affiliations:** 1 Psychiatry & Psychology Research Center, Roozbeh Hospital, Tehran University of Medical Sciences, Tehran, Iran.; 2 School of Nutritional Sciences and Clinical Dietetics, Tehran University of Medical Sciences, Tehran, Iran.; 3 Department of Biostatistics and Epidemiology, School of Public Health, Tehran University of Medical Sciences, Tehran, Iran.; 4 Iran University of Medical Sciences, Gastrointestinal & Liver Diseases Research Center, Tehran, Iran.; 5 Islamic Azad University, Science and Research Branch, Tehran, Iran.

**Keywords:** *Appetite*, *Compulsive Eating Scale (CES)*, *Eating Disorder*, *Obesity*, *Overweight*, *Women*

## Abstract

**Objective:** Compulsive or binge eating is a kind of disturbed eating behavior, which is mostly observed among dieting women, and is integrated with appetite disorder, and uncontrolled eating of plenty of junk food. The Compulsive Eating Scale (CES) created first by Kagan & Squires in 1984, is an eight-item self-reporting instrument that is made to measure the severity of binge eating disorder. The aim of this study was to provide the reliability and validity of the Persian version of Compulsive Eating Scale (CES) among overweight and obese women in Iran.

**Method:** One hundred and twenty six (N = 126) overweight and obese women consented to participate in this study. We estimated the anthropometric indices, including body weight, height, waist and hip circumferences, a total body fat percentage, and visceral fat level with body analyzer all in standard situations. Then, the participants completed the CES. Next, to assess concurrent validity, Beck Depression Inventory, Spielberger anxiety scale, appetite visual analogue rating scale, Food Craving questionnaire, Three-Factor Eating Questionnaire-R18, and Restraint eating visual analogue rating scale were performed simultaneously. To assess test-retest reliability, CES was repeated for all the participants two weeks later. Moreover, we reported the internal consistency and factor analysis of this questionnaire. Furthermore, we estimated the concurrent correlation of CES with logically relevant questionnaires and body composition and anthropometric indices.

**Results:** Based on the reliability analysis and factor analysis of the principal component by Varimax rotation, we extracted two factors: eating because of negative feelings, and overeating. Internal consistency (Cronbach's alpha) of the CES was 0.85 (Cronbach alpha of the factors was 0.85, and 0.74, respectively). The test-retest correlation of the CES was 0.89. Also, the split-half reliability of the questionnaire was established with the correlation coefficient between Sets I and II. The correlation was 0.85.

**Conclusion**: This study provides preliminary support for the reliability and validity of the Persian version of the CES. This instrument would be helpful in measuring the clinical practice and research studies of obesity, appetite and eating disorders reliably and validly.

Commpulsive or binge eating is a type of disturbed eating behavior, which is integrated with appetite disorder, and uncontrolled eating of plenty of junk food (1). The prevalence is much more in women, especially those seeking weight reduction (2). People with compulsive eating disorder usually overeat because they are feeling lonely, bored and depressed. They also fail to stop overeating sugary and high fat foods. Hence, it is highly connected with overweight or obesity situations (3). Furthermore, long-term treatment of obesity is connected with efficient and long-term treatment of binge eating disorder. In addition, the possibility of other psychiatric comorbidities is much more in obese patients with compulsive eating disorder (2). Some social and psychological factors may affect eating behaviors, including feeling lonely, feeling blue or anxious. Particularly, teenagers and women may eat more unhealthy foods when they are bored, alone or depressed. Binge eating disorder can also be seen in the context of borderline personality disorder, atypical depression and bipolar disorder. Thus, both diagnoses can be given if full criteria are met. Furthermore, if exists, the severity of comorbid depression and appetite disorders are associated with the severity of binge eating (2). Differential diagnosis with bulimia nervosa is night eating (4) and that patients with overeating do not try to compensate for binge eating by purging the behaviors, dieting, and laxatives (5, 6). A valid diagnosis of binge eating disorder with a valid instrument in a clinic is highly important because treatment protocols and patients’ response are different from bulimia nervosa (2). 

Reliable and valid instruments are needed in clinical practice and in research to evaluate and interpret subjective measurements such as appetite and eating disorders. Within the field of eating disorders, a large number of questionnaires have been developed worldwide, with acceptable to good psychometric properties. Nonetheless, lack of valid and reliable Persian tools to assess eating habits in clinical setting and research is evident. This reveals the importance and reason to perform reliability and validity studies. 

The Compulsive Eating Scale (CES) created first by Kagan & Squires (7) in 1984, is an eight-item self- reporting instrument that is made to measure the severity of binge eating disorder. It evaluates the failure to control one’s eating behavior. Two factors of “overeating” and “eating when not hungry” have been derived from this instrument (8, 9). Scores are ranging from 8 to 40, with higher scores showing more sever being eating disorder. This instrument was first developed to measure patterns of unrestrained eating behavior in high school students. The items of questionnaire were as follows: “Eating because of feeling lonely”, “feeling completely out of control when it comes to food”, “eating so much that stomach hurts”, “eating because of being upset or nervous”, “eating too much because you are bored”, “Getting out of bed just to go to the kitchen and finish the rest of some delicious food because you know it was there,, “eating so much food so fast that you don't know how much you ate or how it tasted”, “going out with friends for the purpose of overstuffing yourself with food”. The answering choices were to circle the one of the following responses: “A- Never; B- Once or twice a year; C- Once a month; D- Once a week; E- More than once a week” (10). Cronbach’s alpha of 0.75 shows an acceptable internal consistency in school students (8). Due to the content of the instrument and the characteristics of eating disorder, its application has also been expanded into clinical practice. When the application of instruments is developed to a new target population (for example new gender or age group or new condition), its reliability and validity should be reassessed in that new population. Furthermore, this instrument is reported to be valid and reliable in assessing binge eating disorder in the student samples in Western societies, but because of culture related differences between the Western and Eastern countries, we decided to test its reliability and validity in Persian overweight or obese women for the first time.

The aim of this study was to assess the psychometric properties, the reliability and validity of the Persian version of the compulsive eating scale (CES) in overweight and obese women and its relationship with some body composition and dietary intake variables.

## Materials and Method

***Participants***

The participants were 126 overweight and obese women who referred to a weight reduction clinic. Before starting a weight loss program they were invited to participate in this study. The study protocol was described to them and informed consent was obtained. Anthropometric assessments were performed by a clinical dietitian. Anthropometric indices, including body weight, height, BMI, waist and hip circumferences, and total body fat percentage, and visceral fat level were estimated with body composition analyzer all in standard situations. Then participants were asked to fill the Persian version of the CES, Beck Depression Inventory, Spielberger Anxiety Scale, Appetite Visual Analogue Scale, Food Craving Questionnaire, and the Three-Factor Eating Questionnaire-R18. Furthermore, participants filled a three-day food record. Two weeks later, the tests repeated for 126 participants in similar situations.

***Instruments***

***Compulsive Eating Scale (CES):*** We aimed to measure its reliability and validity in this study.

***Beck Depression Inventory (BDI):*** This instrument is a questionnaire consisting of 21 self-report questions to measure depressive symptoms in adults and adolescents over 13 years. The questionnaire is designed based on the criteria of the DSM for evaluating the severity of symptoms of major depressive disorder. Psychometric properties of the Persian version of BDI reveal that items and factors are reliable and valid to detect and evaluate depression, and its results are matched with earlier instruments and with the results of the structured diagnostic interview. The internal consistency of the Persian version of BDI was very well (11).

***Spielberger Anxiety Scale:*** The reliability and validity of this questionnaire has been evaluated around the world, including Iran. The reliability and validity of the Persian version of Spielberger anxiety scale has been approved by Cronbach's alpha coefficient of around 0.90 (12).

***Appetite Visual Analogue Scale:*** This instrument is a grading tool to quantify the subjective variables such as appetite (13). Visual analogue scale for appetite had acceptable concurrent validity with calories intake from the diet, body weight, body mass index, and body fat percentage (13-15).

***Three-Factor Eating Questionnaire-R18:*** This test is a widely used questionnaire in surveys of eating behavior. The psychological characteristics, and validity and reliability of the questionnaire had been evaluated and approved previously. It has good internal consistency (α = 0.73), and test-retest reliability (r = 0.87) (15).

***Food Craving Questionnaire (FCQ):*** This instrument is another widely used questionnaire for assessing eating behavior. The psychological characteristics, and validity and reliability of the Persian version of this tool had been evaluated and approved previously, showing an excellent internal consistency (α = 0.90) and test-retest reliability (r = 0.92) (14).

***Body composition Analyzer: ***We used Omron HBF-500 BIA (Omron Co., Japan) device which, involves eight electrodes: Tetrapolar electrodes in footpads, and another 4 set of electrodes in the handle. The participants stood on the metal footpads in bare feet and grasped a pair of electrodes fixed to a handle with arms extended in front of the chest. This instrument assesses total body fat, visceral fat, lean body mass, and basal metabolic rate as well as body weight and BMI. The clinical validity of this instrument in measuring body composition is already approved, compared with Dual-Energy X-Ray Absorptiometry and Magnetic Resonance Imaging (MRI) (16).

***Weight and Height Gage***

***Non-Stretchable Tape***

***Procedure***

The CES was translated into the Persian by the first author (SA.M). Then, the third author who is a psychiatrist and native in Persian and fluent in English (MR.M) confirmed the accuracy of the translation and content validity of the Persian version of CES. Then, an independent translator back translated CES into English. The identical content of the two versions was confirmed by a language expert. Also, the face validity of the questionnaire was checked by all authors, and content validity was compared to DSM-5 criteria for eating disorder (2), and they were good. Then, 200 overweight or obese women were invited and consented to participate, and 126 finished the study procedure successfully. 

***Data Analysis***

Descriptive statistics were used to describe the participants, and the Cronbach’s Alpha was used to assess its internal consistency. Factor analysis was performed to determine the dimensions and naming the factors of the questionnaire. Pearson correlation coefficient was used to assess the test–retest reliability. We also assessed the correlations between CES and Beck Depression Inventory, Spielberger Anxiety Scale, Appetite Visual Analogue Scale, and Three- Factor Eating Questionnaire-R18, weight, body fat and waist circumference. All the analyses were performed, using PASW Statistics 18, Release Version 19.0.0 (SPSS, Inc., 2009, Chicago, IL, ww.spss.com). Significance was set at p < 0.05.

## Results

Participants were 18-60 years, overweight or obese women with the mean age of 40.5±10.2 years. Demographic Characteristics of the Participants are presented in table 1. 

**Table1:** Demographic Characteristics of the Participants

	**N** *	**Percent**
**Obesity start age**		
Before puberty After puberty	16 109	12.8 87.2
**Education**		
Diploma or lower Associate degree or BA Postgraduate	76 40 9	60.8 32 7.2
**Socioeconomic status**		
Low Middle High	9 111 5	7.2 88.8 4
**Marital status**		
Single Married Widowed or divorced	20 102 3	16 81.6 2.4
**Child delivery number**		
0 1 2 3 4 5	25 17 44 34 4 1	20 13.6 35.2 27.2 3.2 0.8

* Total: 125 (One Case Missing)

**Table2:** KMO and Bartlett's Test

Kaiser-Meyer-Olkin Measure of Sampling Adequacy		0.85
Bartlett's Test of Sphericity	Approx. Chi-Square	524.672
	df	28
	Sig.	<0.0001

***Factor Analysis***

We used factor analysis to analyze the construction of the questionnaire and naming the factors. We benefited from the principal-components analysis to extract the factors by varimax rotation. We used the rotation method since we supposed that the factors were correlated with each other or the components were not independent of each other. Various investigators have proposed that if a factor explains 5% or more of the total variance, the factor is significant (19).

**Figure1:**
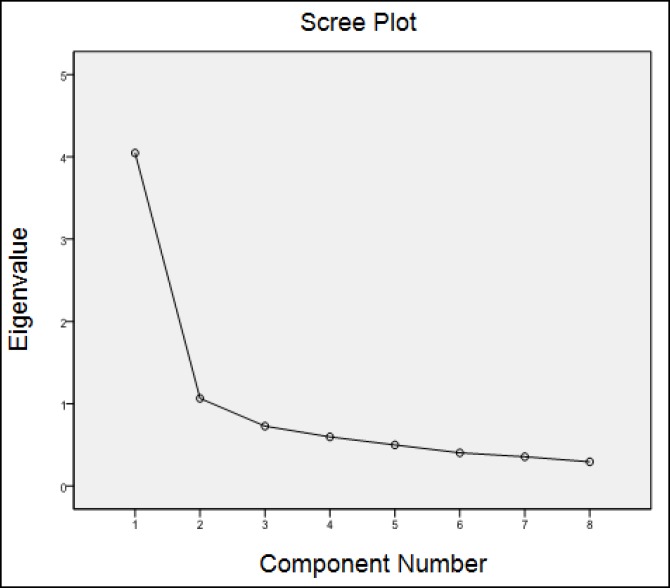
Factors Extracted by Scree Plot

The Kaiser–Meyer–Olkin (KMO) index for the sampling adequacy was 0.85 (Bartlett’s test of sphericity was significant at P<0.0001; df = 28). Therefore, rejection of the null hypothesis that the variables used in the analysis were not correlated in the studied population was allowed. Furthermore, based on the Kaiser–Meyer–Olkin (KMO) index factor analysis was allowed via correlation matrix (Table 2). The range of factor loadings for the items and the variances as well as the Eigen values are presented in Table 3; Eigen values of greater than 1.00 explained variance of 63.9%. Factor analysis via scree plot is shown in Figure 1. 

**Table3:** Total Variance Explained

	**Extraction Sums of Squared Loadings**			**Rotation Sums of Squared Loadings**		
**Component**	**Total**	**% of Variance**	**Cumulative %**	**Total**	**% of Variance**	**Cumulative %**
1	4.047	50.589	50.589	2.946	36.822	36.822
2	1.066	13.328	63.917	2.168	27.095	63.917

**Table4:** Rotated Component Matrix^a^

	**Component**	
	**1**	**2**
c1. Eating because of feeling lonely	0.716	
c2. Feeling completely out of control when it comes to food	0.677	
c3. Eating so much that stomach hurts	0.612	0.504
c4. Eating because of feeling upset or nervous	0.838	
c5. Eating too much because you are bored	0.854	
c6. Going out with friends for the purpose of overstuffing yourselves with food		0.806
c7. Eating so much food so fast that you don't know how much you ate or how it tasted		0.643
c8. Getting out of bed, and going into the kitchen to finish the rest of some delicious food because you know it was there		0.775

Extraction Method: Principal Component Analysis Rotation Method: Varimax with Kaiser Normalization

a . Rotation Converged in 3 Iterations

**Table5:** Inter-Correlations among the CES Scores and Body Weight, Waist Circumference, BMI, Fat Percentage, and Muscle Percentage for 125 Participants

	**CES**	
	**Pearson Correlation**	**Sig (2-Tailed)**
Weight	0.167	0.060
Waist Circumference	0.217	0.016
BMI	0.132	0.142
Fat%	0.156	0.090
Muscle %,	-0.09	0.300
Visceral fat	0.015	0.860

**Table6:** Inter-Correlations among the CES Scores, BDI, Appetite Visual Analogue Scale, Spielberger Anxiety Scale, Food Craving Questionnaire, Three-Factor Eating Questionnaire-R18, Restraint Eating Visual Analogue Scale (TFEQ-R18 Subscale)

	**CES**	
	**Pearson Correlation**	**Sig (2-Tailed)**
Beck Depression Inventory (BDI)	0.48	<0.0001
Appetite Visual Analogue Scale	0.63	<0.0001
Spielberger Anxiety Scale	0.39	<0.0001
Food Craving Questionnaire	0.65	<0.0001
Three-Factor Eating Questionnaire-R18 (TFEQ-R18)	0.62	<0.0001
Restraint Eating Visual Analogue Scale (TFEQ-R18 Subscale)	-0.56	<0.0001

**Table7:** Inter-Correlations among the CES Scores, Dietary Intake of Carbohydrates, Fat, Protein, and Calorie

	**CES**	
	**Pearson correlation**	**Sig (2-tailed)**
Calories	0.16	0.098
Carbohydrates	0.298	0.002
Fat	-0.208	0.034
Protein	0.162	0.100
Sugar	0.265	0.007

Two components were loaded on the expected factors. Factor 1 was stronger, explaining the greater percentage of the variance (50.5%); five items were loaded on this factor, and the factor (Table 4) was named “eating because of negative feelings”, and the second factor “Overeating”, which explained 13.3% of the variance. We have presented the results of factor analysis with varimax rotation on the eight items of the CES in Table 4.

***Analysis of Internal Consistency of the CES***

The internal consistency of the CES was well accepted. The reliability of the CES as measured by Cronbach’s alpha coefficient was 0.85. (Cronbach alpha of the factors was 0.85, and 0.74, respectively). Overall, the results revealed a high level of internal consistency for the eight items, and they were homogenous. Moreover, none of the items had to be deleted to improve α.

***Test-Retest and Split Half Reliability***

The test–retest reliability of the questionnaire was administered to all the participants with a two weeks interval, and revealed a significant level of 0.001. Pearson correlation showed a consistency of 0.89 between the two administrations. 

In addition, we performed the split-half reliability analysis for the CES. The correlation coefficient between Sets 1 and 2 was 0.85. Statistical significance was set at 0.05 levels.

***Validity of the CES***

***Concurrent Validity***

Inter-correlations among the CES scores, body composition and anthropometric indices are shown in Table 5.

Inter-correlations among the CES scores, BDI, appetite and restrain visual rating scale are shown in Table 6.

Inter-correlations among CES scores dietary intake of carbohydrate, fat, protein and calorie are shown in Table 7.

The effect size of the correlation between the CES scores and anthropometric indices, BDI, appetite visual scale, and dietary intake provided evidence for the concurrent validity of the CES.

## Discussion

This was the first study to report the reliability and validity of compulsive eating scale (CES) in an Iranian culture and in overweight and obese women. This instrument was designed to measure the severity of binge eating disorder. It evaluates emotional eating and the failure to control eating behavior. Two factors of “overeating” and “eating when not hungry” have been derived from the English version of this instrument (8, 10). Similarly, in the Malaysian version of CES also the two-factor construction accounted for 51.0% of the variance. Factor loading ranged from 0.41 to 0.50(9). Likewise, in our study, we loaded two expected factors. Factor 1 was stronger, explaining the greater percentage of the variance (50.5%); five items were loaded on this factor and the factor was named “eating because of negative feelings”. The second factor derived was named “over-eating”, which explained 13.3 % of the variance. Rotated Component Matrix and the concept of the items with grater variance were effective in naming the two factors (items number 5 and 6). Furthermore, we named the first factor differently because we thought eating was more frequently a response to unpleasant or negative emotional states. In fact, compulsive eaters eat more food to cope with anxiety, reduce tension and feel better.

Our findings indicated that the internal consistency of the Persian version of the CES and its subscales is acceptable to excellent, which is in line with the internal consistency of the English version of the scale in school age children, with Cronbach’s alpha of 0.75 (7, 8). Similarly, in the Malaysian version of CES, Ganasegeran and colleagues have reported the Cronbach’s alpha coefficient of 0.80 among university students (9). Kusum Lata Mishra, and colleagues in a study on college students in Oman reported an excellent internal consistency of the CES with Cronbach’s alpha of 0.81(17). Consequently, these findings are all in line with our results and prove the suitable internal consistency of the different versions of the CES.

Brittany Gower and colleagues (18) measured the relationship between CES and the stressful situations on 82 male and female traditional full-time college students aged 18 to 23. They found a significant positive correlation between CES and stressful situations, indicating that as stress increased for the male and female students, compulsive eating also increased. Azza M. and colleagues assessed the correlation between stress and CES among nursing students in Egypt. They found a highly significant positive correlation between CES and stress (10). Kusum Lata Mishra and colleagues in a study on college students in Oman also reported a significant positive correlation between CES and stress. The present data are consistent with earlier studies, which suggest that as stress or anxiety increases, food consumption also increases. The only difference was that we performed our study just on overweight or obese women, but obtained the same results. Because of the mutual effects of nutritional factors and depression (19,20) we hypothesized , in the beginning of the study, that anxiety, depression and appetite may influence uncontrolled eating, and these may be positively correlated. Ghiz l. and colleagues (3) reported that CES has a significant positive correlation with BDI, and Three Factor Eating Questionnaire (TFEQ) in women. Furthermore, Ondercin and colleagues reported that women with higher score of the CES ate more often in response to emotional states such as anxiety, boredom, loneliness, and depression (21). In our study, positive significant correlations among CES scores, BDI, Appetite Visual Analogue Scale, Spielberger Anxiety Scale, Food Craving questionnaire, Three-Factor Eating Questionnaire-R18, as well as the negative correlation between CES and Restraint eating Visual Analogue Scale (TFEQ-R18 Subscale where we logically expected) demonstrate acceptable convergent and divergent validity. The results of our study imply that the more anxiety, apprehension, and depression an overweight or obese woman feels, the more likely she is to eat emotionally.

In our study, CES was positively correlated with body weight, BMI, waist circumference, total body fat percentage and visceral fat (convergent validity), but negatively correlated with muscle percentage (divergent validity). Nevertheless, none of these correlations were significant. These weak correlations revealed some moderator variables between compulsive eating and body weight or total body fat percentages. Some direct variables such as dietary intake and appetite may mediate these correlations. However, here the direction of the correlation was more important. Similarly, Timmerman and colleagues (22) examined the correlation between binge eating, caloric intake, body fat percentage and BMI in non-purge binge eating (i.e., compulsive eating) women. They reported that there was a low, but significant relationship between binge (compulsive) eating severity and BMI. However, the relationship was not significant between binge eating severity and body fat percentage. In addition, other researchers revealed the relationship of anthropometric indices with mood and eating disorders (23-25). All these reports are in line with our results.

What is more, in our study, CES was positively correlated with dietary intake of calories, protein, carbohydrates, and simple sugar, but not fat (negative relationship). This finding is controversial since some sources have reported higher amount of sugary and high fat foods consumption in binge eaters (2). However, others have reported that sugar and fat act differently on the brain receptors, especially pleasure and reward regions, which are associated with compulsive eating behavior. Stice and colleagues (26) reported that sugar may more vigorously influence compulsive eating than fat. Furthermore, Yu, Z. and colleagues reported that in rat models of binge eating, ovarian hormones restrain the fat intake (27). Furthermore, since these obese women are selected beside a weight reduction diet they may have reduced their fat intake prior to the study. Also, Yanovski and colleagues (28) have reported more calorie intake in binge eaters, which is consistent with our results and altogether reveals good concurrent and convergent validity of CES. 

## Limitations

Lack of a concurrent psychometric analysis and limited number of items in the instrument were among the limitations of the study.

## Conclusion

In essence, this study showed that the Persian version of the CES is a psychometrically sound instrument to assess eating psychopathology in Iranian overweight and obese women. However, the results should be interpreted with caution. The nature of the sample (i.e., only clinical sample of overweight and obese women) limit the generalization of the findings into wide-ranging population, especially men. 

## References

[1] Olsen CM (2011). Natural rewards, neuroplasticity, and non-drug addictions. Neuropharmacology.

[2] Association AP (2013). Binge Eating Disorder, DSM-5 Diagnostic Criteria. Diagnostic and Statistical Manual of Mental Disorders.

[3] Ghiz L, Chrisler JC (1995). Compulsive eating, obsessive thoughts of food, and their relation to assertiveness and depression in women. J Clin Psychol.

[4] Grilo CM, Masheb RM (2004). Night-time eating in men and women with binge eating disorder. Behav Res Ther.

[5] Saunders R (2004). "Grazing": a high-risk behavior. Obes Surg.

[6] Pull CB (2004). Binge Eating Disorder. Curr Opin Psychiatry.

[7] Kagan DM SR (1984). Eating disorders among adolescents: patterns and prevalence. Adolescence.

[8] Kagan DM SR (1984). Compulsive eating, dieting, stress and hostility among college students. J Coll Stud Pers.

[9] Ganasegeran K, Al-Dubai SA, Qureshi AM, Al-abed AA, Am R, Aljunid SM (2012). Social and psychological factors affecting eating habits among university students in a Malaysian medical school: a cross-sectional study. Nutr J.

[10] Azza M, Abd el-aziz sas, Yousseria E Yousef (2014). Relationship between Stress and Eating Habits among Nursing Students in Assiut. Med J Cairo Univ.

[11] Ghassemzadeh H, Mojtabai R, Karamghadiri N, Ebrahimkhani N (2005). Psychometric properties of a Persian-language version of the Beck Depression Inventory--Second edition: BDI-II-PERSIAN. Depress Anxiety.

[12] Shahri P (1372). Preliminary Study of Reliability and validity of Spielberger Trait Anxiety Inventory (STAI).

[13] Parker BA, Sturm K, MacIntosh CG, Feinle C, Horowitz M, Chapman IM (2004). Relation between food intake and visual analogue scale ratings of appetite and other sensations in healthy older and young subjects. Eur J Clin Nutr.

[14] Seyed-Ali Mostafavi, Mohammad Reza Mohammadi, Payam Hosseinzadeh, Mohammad Reza Eshraghian, Shahin Akhondzadeh, Mohammad Javad Hosseinzadeh-Attar (2012). Dietary intake, growth and development of children with ADHD in a randomized clinical trial of Ritalin and Melatonin co-administration: Through circadian cycle modification or appetite enhancement?. Iran J Psychiatry.

[15] Seyed-Ali Mostafavi, Mohammad Reza Mohammadi, Mohammad Reza Eshraghian, Saeed Hosseini, Fahimeh Ahmadi moghadam, Payam Hosseinzadeh (2016). Psychometric Properties, reliability and validity of Persian Version of the Three-Factor Eating Questionnaire-R18 (TFEQ-R18) in overweight and obese women and its Relationship with Some body composition and dietary intake variables. IJPS.

[16] Mock M, Ryan ED, Gerstner GR, Tweedell AJ, Kleinberg CR, Hirsch KR (2016). Validity of a Multi-compartment Body Composition Model Using Body Volume Derived from Dual-Energy X-ray Absorptiometry: 3715 Board #154 June 4, 8: 00 AM - 9: 30 AM. Med Sci Sports Exerc.

[17] Kusum Lata Mishra GPM (2013). Establishing Relationship between “Stress” and “Eating” Leading to Overweight among College Students in Sultanate of Oman. Journal of Business and Economics.

[18] Brittany Gower CEH, Zachariah K (2008). Crooks The Relationship Between Stress and Eating in College-Aged Students. URJHS.

[19] Elham Ranjbar, Masoumeh Sabet Kasaei, Minoo Mohammad-Shirazi, Javad Nasrollahzadeh, Bahram Rashidkhani, Jamal Shams (2013). Effects of zinc supplementation in patients with major depression: a randomized clinical trial. Iran J Psychiatry.

[20] Elham Ranjbar, Jamal Shams, Masoumeh Sabetkasaei, Minoo M-Shirazi, Bahram Rashidkhani, Ali Mostafavi (2014). Effects of zinc supplementation on efficacy of antidepressant therapy, inflammatory cytokines, and brain-derived neurotrophic factor in patients with major depression. Nutritional neuroscience.

[21] Ondercinp A (1979). Compulsive eating in college women. Journal of College Student Personnel.

[22] Timmerman GM, Stevenson JS (1996). The relationship between binge eating severity and body fat in nonpurge binge eating women. Res Nurs Health.

[23] Ahmadi SM, Keshavarzi S, Mostafavi SA, Bagheri Lankarani K (2015). Depression and Obesity/Overweight Association in Elderly Women: a Community-Based Case-Control Study. Acta Med Iran.

[24] Ahmadi SM, Mohammadi MR, Mostafavi SA, Keshavarzi S, Kooshesh SM, Joulaei H (2013). Dependence of the geriatric depression on nutritional status and anthropometric indices in elderly population. Iran J Psychiatry.

[25] SA Mostafavi, S Hosseini (2014). Weight Management, Energy Metabolism, and Endocrine Hormones-Review Article. Iranian Journal of Public Health.

[26] Stice E, Burger KS, Yokum S (2013). Relative ability of fat and sugar tastes to activate reward, gustatory, and somatosensory regions. Am J Clin Nutr.

[27] Yu Z, Geary N, Corwin RL (2008). Ovarian hormones inhibit fat intake under binge-type conditions in ovariectomized rats. Physiol Behav.

[28] Yanovski SZ, Sebring NG (1994). Recorded food intake of obese women with binge eating disorder before and after weight loss. Int J Eat Disord.

